# 

**Published:** 1889-04

**Authors:** 


### PAMPHLETS RECEIVED.

Diseases of the Nose and Pharynx and Their Treatment. By W. Cheatham, M.D.

Malaria and the Causation of Periodic Fever. By Henry B. Baker, M.D.

The Philosophy of Memory. By D. T. Smith, M.D.

The President's Annual Address. By Robert Battey, M.D.

On the Circular Polarizations of Certain Tartrate Solutions. I. By J. H. Long.

New Form of Posterior Colporrhaphy. By. J. H. Kellogg, M.D.

Experimental Researches Respecting the Relation of Dress to Pelvic Disorders of Women. By J. H. Kellogg, M.D.

Report of Forty-eight Cases of Alexander's Operation. By J. H. Kellogg, M.D.

The Etiology of Dipsomania. By Lewis D. Mason, M.D.

The Radical Cure of Varicocele. By Morris H. Henry, M.A., M.D.

Extremes in Altitude in Southern California. By Walter Lindley, M.D.

Transactions of the American Otological Society. Vol. 4, Part 2. 1888.

Annual Report of the Supervising Surgeon-General of the Marine Hospital Service of the United States for the Fiscal Year 1888.

Pulmonary Consumption Considered as a Neurosis. By Thomas J. Mays, M.D.

Two Cases of Gunshot Wound of the Abdomen. By N. Senn, M.D.

Inflation of the Stomach with Hydrogen Gas in the Diagnosis of Wounds and Perforations of this Organ. By N. Senn, M.D.

The Failure of Dr. J. B. Thomas' Treatment of Urethral Stricture by Electrolysis. By Robert Newman, M.D.

The Treatment of Peritonitis by Abdominal Section. By L. S. McMurtry, M.D.

Case of Typhlitis with Double Perforation of the Cæcum, and Peritonitis. By L. S. McMurtry, M.D.

Electricity vs. Tait, or The Use of Electricity in Inflammations as Found in Gynecology. By George F. Hurlburt, M.D.

Preliminary Report of an Operation for the Formation of an Artificial Pupil through the Sclerotic Coat of the Eyeball. By George Strawbridge, M.D.

The Influence of Cocaine upon Ophthalmic Surgery. By Samuel Theobald, M.D.

On Some Mild Measures in the Treatment of Intra-Nasal Hypertrophies and Inflammations. By W. H. Daly, M.D.

Is Astigmatism a Factor in the Causation of Glaucoma? By Samuel Theobald, M.D.

The Histology and Surgical Treatment of Uterine Myoma. By Henry O. Marcy, M.D.

The Climate of the Southern Appalachians. By Henry O. Marcy, M.D.

Placental Development. By Henry O. Marcy, M.D.

The Case of the Emperor Frederick. Official Reports. By Henry Schweig, M.D.

The Anatomy and Pathology of the Thymus Gland. By A. Jacobi, M.D.

How Microörganisms Enter the Body. By George N. Kreider, M.D.

Femoral Osteotomy for the Correction of Deformity Resulting from Hip-Joint Disease. By Ap. Morgan Vance, M.D.

Cases in Orthopœdic Surgery. By Ap. Morgan Vance, M.D.

Pregnancy and Operative Surgery. By Louis McLain Tiffany, M.D.

Osteotomy for Anterior Curves of the Leg. By DeForrest Willard, M.D.

The Electrolytic Decomposition of Organic Tissues. By George H. Rohe, M.D.

Food vs. Bacilli in Consumption. By Ephraim Cutler, M. D., LL.D.

Pressure Forceps, vs. the Ligature and the Suture in Vaginal Hysterectomy. By E. C. Dudley, M.D.

Laparotomy for Ascites. By Thomas A. Ashby, M.D.

Pathology and Treatment of Alopecia Areota. By A. R. Robinson, M.B.

The Question of Relationship between Lichen Planus (Wilson) and Lichen Ruber (Hebra). By A. R. Robinson, M.B.

Biniodide of Mercury. Its Antiseptic Use. By Eugene P. Bernardy, M.D.

Intubation in Chronic Stenosis of the Larynx. By Joseph O. Dwyer, M.D.

Intubation of the Larynx in Diphtheritic Croup. By Dillon Brown, M.D.

The Science of Successful Surgery. By John B. Roberts, M.D.

Emergency Hospitals. By Hal C. Wyman, M. S., M.D.

Twenty-Eighth Annual Report of the Cincinnati Hospital. 1888.

---

### MISCELLANY.

---

***Seventh Decennial Convention for Revising the Pharmacopœia of the United States.*** —Notice is hereby given that, in accordance with and by virtue of the authority vested

Volume LVIII.] APRIL, 1889. [Number 4.

### The CHICAGO MEDICAL JOURNAL AND EXAMINER

EDITOR:

S. J. JONES, M.D., LL.D.

COLLABORATORS:

CHICAGO:

Professor E. ANDREWS, M.D., LL.D.
Professor J. BARTLETT, M.D.
Professor W. T. BELFIELD, M.D.
Professor F. BILLINGS, M.D.
Professor W. H. BYFORD, A.M., M.D.
Professor L. CURTIS, A.M., M.D.
Professor N. S. DAVIS, Jr., A.M., M.D.
Professor O. C. DE WOLF, A.M., M.D.
Professor C. FENGER, M.D.
Professor J. N. HYDE, A.M., M.D.
Professor E. F. INGALS, A.M., M.D.
Professor F. S. JOHNSON, A.M., M.D.
Professor H. A. JOHNSON, M.D., LL.D.
Professor C. W. PURDY, M.D.
Professor C. G. SMITH, A.M., M.D.
Professor E. WING, A.M., M.D.
Professor E. C. DUDLEY, A.B., M.D.

MILWAUKEE, WIS.:
Professor N. SENN, M.D., Ph.D.

WASHINGTON, D. C.:
S. O. RICHEY, M. D.

UNITED STATES ARMY:
Surgeon D. L. HUNTINGTON, M.D.

UNITED STATES NAVY:
Surgeon-General J. M. BROWNE, M.D.
Medical-Director A. L. GIHON, A.M., M.D.

MARINE HOSPITAL SERVICE:
Surgeon S. T. ARMSTRONG, M.D., Ph.D.

